# Supplementary material for: Viral load care of HIV-1 infected children and adolescents: A longitudinal study in rural Zimbabwe
Source: PLoS One. 2021 Jan 14;16(1):e0245085. doi: 10.1371/journal.pone.0245085 (PMC7808638; doi:10.1371/journal.pone.0245085)
Supplement: Attachment — Submitted filename: Review of PONE-D-20-20773, HM Hanauske-Abel.pdf [file pone.0245085.s002.pdf]

Review of manuscript

PONE-D-20-20773 by Mapangisana / Katzenstein :

Viral load-differentiated care of HIV-1 infected children and adolescents – a prospective longitudinal study in rural Zimbabwe.

This manuscript addresses a key issue in current HIV service delivery that has globally emerged as an essential topic due to limited resources and stressed or decreasing funding: Viral load-differentiated care (e.g. [1-3]).

To ensure the manuscript's – I think: valuable - contribution to this key issue, the authors should consider the following points that distract from their work and thus require correction, even before further review of the manuscript's methodological and medical aspects:

1. It is disturbing to see the authors classifying themselves into two separate groups that, though *apart* for unspecified reasons, nevertheless state each had contributed *equally* to the manuscript:

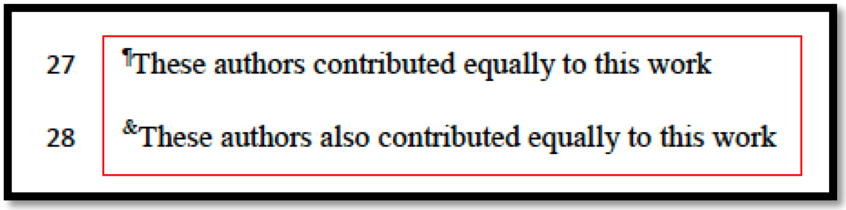
27 ¶These authors contributed equally to this work
28 &These authors also contributed equally to this work

Are the unspecified contributions of the first-named ¶group (line 27) '*separate but equal*' to those of the second-named &group (line 28) and therefore identical with the once legal principle of racism and discrimination in the United States ? That principle has been discredited. Or is the first-named ¶group claiming to be '*more equal*' than the second-named &group, reminiscent of Orwell's *Animal Farm* ?

Disturbingly, not a single one of the authors in the first-named ¶group states that their primary affiliation is anchored at an institution in Zimbabwe.

In the second-named &group of 10 authors, by contrast, those with Zimbabwean names and / or institutional affiliations predominate by a ratio of 9/1.

This separation into two classes of authors is evident on the title page. The authors **<u>must</u>** find a way to put an end to such apartheid.

I suggest they follow PLOS convention and specify select categories of contributions, e.g. similar to the ones in ref. [2]:

Author Contributions

Concept and design: (names)
Data collection and formal data analysis: (names)
Methodology: (names)
Supervision: (names)
Writing original draft: (names)
Writing review & editing: (names)
etc.

2. The title of PONE-D-20-20773 specifically states that this is a "*prospective longitudinal study*" that ran from 2016 to 2018.

On close reading, in particular of the Methods section on '*HIV VL testing*', it appears that the 2018 segment of the study rests entirely on data obtained as part of CBART (Community Based Virus Load Differentiated Care in Rural Africa). But CBART did not exist in 2016, the initial year of the findings reported in PONE-D-20-20773. How then might it be possible that a two-year "*prospective longitudinal study*" is IRB-approved in 2016, at its inception, yet uses clinical protocol and data of CBART, which started years later, on February 1, 2018. From a 2016 IRB perspective, CBART represents an extrinsic and later protocol. This fact, in turn, might raise the added complication of post-hoc analysis of data, up to and including the possible prospect that PONE-D-20-20773 itself could, from its 2016 start, not be a strictly true and genuine "*prospective longitudinal study*".

The Methods section on '*Ethics approval and consent to participate*' does neither address nor resolve the conundrum of how the 2018 CBART dovetails with the 2016 IRB approval of PONE-D-20-20773. Rather, the formal introduction of two different IRB approvals - "*by the Medical Research council of Zimbabwe (MRCZ/A/2269) and by the Biomedical Research Training Institute IRB (AP143/2018)*" – further enhances this conundrum: Where participants enrolled in MRCZ/A/2269 **and** in AP143/2018 ? Did all the patients / parents sign two different assent /consent forms ?

An attentive reader is left with the impression that PONE-D-20-20773 might have been skillfully tiled and assembled from different protocols. If this should be so, this fundamental ethics issue might be resolved properly by, and might require, an IRB approval for human data use.

To address this matter objectively, I suggest that the currently unavailable and inaccessible IRB approvals MRCZ/A/2269 and AP143/2018 are uploaded to the PLOS ONE submission site for review. They are not disclosed at this time, just cited.

Such an IRB approval for 'data tiling and compiling' will leave another conundrum unresolved, however: The CBART study, which supposedly completed in February 2020, specifically defines the identification of "*drug resistance mutations*" as one of its two Secondary Outcome Measures, the other one being the "*Number of participants with confirmed virology failure who switched regimens. Change to second line regimen after confirmed virological failure*" (see: https://clinicaltrials.gov/ct2/show/NCT03986099).

Yet PONE-D-20-20773, which rests on that very CBART study, states "*that drug resistance testing was not performed on patients failing ART* " (line 351). The authors themselves instantly recognize this to be "*a limitation of the study* " (line 350).

The authors leave utterly unmentioned that mutation analysis is integral to CBART. This raises questions: Why do they not use CBART data on "*drug resistance mutations*", available at least per protocol, in a manner that renders PONE-D-20-20773 as strong scientifically as it could and should be ? Are certain CBART data too good for PONE-D-20-20773 ? Is it really the authors' choice to submit to PLOS ONE a manuscript for publication that, in their words, is weakened by their own choosing ?

The authors must resolve these conundrums.

3. Per the WHO HIV Treatment Guidelines and academic publications (see [2]), a core aspect of viral load-differentiated care is to "both serve the needs of PLHIV (*people living with HIV*) better and reduce unnecessary burdens on the health system" [1], to "reduce the frequency of clinic visits for patients stable on ART" with "anticipated reductions in the costs of clinic visits, due to these being less frequent for many patients"[2]. Consequently, "differentiated care models could decrease health systems costs in 38 countries in sub-Saharan Africa" [3].

   This decisive aspect of viral load-differentiated care is entirely absent from PONE-D-20-20773, and its focus on just viral load management makes the study almost a description of routine clinical decisions – clearly, every doctor entrusted with the care of HIV patients will adjust the ARV regimen in response to viral load.

   By focusing on viral load-differentiated care, the authors have given themselves the burden of showing more than just viral load responses, and they have to answer simple questions like '*Did the patients who responded to viral load-differentiated care in actual fact require fewer clinic visits, fewer medical resources, and did they have fewer days lost at school, fewer hours lost on the way to clinic, fewer hours wasted while waiting to be seen by the medical team ?*' If the authors can create a clinic-based quantitative monetary parameter, even better (e.g. costs, '*how many bandages used?*', etc.)

   I respectfully submit it might be possible to read some of the answers to such questions, essential to viral load-differentiated care, off the medical charts of enrolled patients.

   The inclusion of such data will turn this manuscript into a reality-based contribution to the discussion on resource utilization in viral load-differentiated care. I encourage the authors to make that effort.

4. I note a number of the usual diligence issues that reviewers like to discover as evidence of their own diligence, such as spelling and capitalization (e.g. line 10: 'Cape town'), bringing references up to date and into PLOS format (e.g. ref. 12 is one of many that lack the specified format), improving the pixilation of images (e.g. Fig. 1 lacks the proper resolution), and clarifying graphics (e.g. Fig. 2: Why were patients with VL < 1000 switched to 2nd line ? Why were patients with VL ≥ 1000 maintained on 1st line ?). These issues should be addressed.

In summary, this manuscript contains the core of a valuable contribution that deserves the effort of a **Major Revision**.

I recognize and respect the effort especially of the Zimbabwe-based authors, effort that shines through in sentences like "*Whole blood samples were transported to Harare (300 km one-way trip) within 24 hours, and plasma was separated and kept frozen at -20°C* ". Such dedicated front-line research, conducted despite the most horrendous economical deprivation and the most degrading political conditions, is indispensible in the fight against HIV-1 and must be promoted and nurtured.

I encourage the authors to correct the issues identified above and to resubmit an accordingly revised version of their manuscript.
That revised version must meet all PLOS format requirements. Thank you.

References:

1. Grimsrud A, Bygrave H, Doherty M, Ehrenkranz P, Ellman T, Ferris R, et al. (2016) Reimagining HIV service delivery: the role of differentiated care from prevention to suppression. J Int AIDS Soc 19: 21484.
2. Revill P, Walker S, Cambiano V, Phillips A, Sculpher MJ (2018) Reflecting the real value of health care resources in modelling and cost-effectiveness studies-The example of viral load informed differentiated care. PLoS One 13: e0190283.
3. Grimsrud A, Barnabas RV, Ehrenkranz P, Ford N (2017) Evidence for scale up: the differentiated care research agenda. J Int AIDS Soc 20: 22024.
