# Supplementary material for: Viral load care of HIV-1 infected children and adolescents: A longitudinal study in rural Zimbabwe
Source: PLoS One. 2021 Jan 14;16(1):e0245085. doi: 10.1371/journal.pone.0245085 (PMC7808638; doi:10.1371/journal.pone.0245085)
Supplement: Attachment — Submitted filename: Response to reviewers.docx [file pone.0245085.s003.docx]

**Response to reviewers’ comments**

1. Please ensure that your manuscript meets PLOS ONE's style requirements, including those for file naming. The PLOS ONE style templates can be found at

<https://journals.plos.org/plosone/s/file?id=wjVg/PLOSOne_formatting_sample_main_body.pdf> and<https://journals.plos.org/plosone/s/file?id=ba62/PLOSOne_formatting_sample_title_authors_affiliations.pdf>

**Response: The authors have ensured that the manuscript meet PLOS ONE requirements**

2. We note that Figure 1 in your submission contain map images which may be copyrighted. All PLOS content is published under the Creative Commons Attribution License (CC BY 4.0), which means that the manuscript, images, and Supporting Information files will be freely available online, and any third party is permitted to access, download, copy, distribute, and use these materials in any way, even commercially, with proper attribution. For these reasons, we cannot publish previously copyrighted maps or satellite images created using proprietary data, such as Google software (Google Maps, Street View, and Earth). For more information, see our copyright guidelines: <http://journals.plos.org/plosone/s/licenses-and-copyright>. We require you to either (1) present written permission from the copyright holder to publish these figures specifically under the CC BY 4.0 license, or (2) remove the figures from your submission:

**Response: Figure 1 was constructed from available caption/map from the Ministry of Health Zimbabwe database. Hence, it is similar but not identical to the original image as it contains the specific study sites inserted by the authors. The authors have specified this in the Figure 1 caption.**

3. Thank you for stating the following in the Financial Disclosure section:

"Funding for the study was provided as Investigator Sponsored Research (ISR-17-10142) from Gilead Sciences Inc., Foster City Ca. TM and RM received support from the Fogarty International Centre of the National Institutes of Health (D43TW010547). TM was supported by DELTAS Africa Initiative SSACAB (Grant No. 107754/Z/15/Z) of the African  Academy of Sciences (AAS) Alliance for Accelerating Excellence in Science in Africa (AESA), New Partnership for Africa’s Development Planning and Coordinating Agency (NEPAD Agency) and the Welcome Trust (Grant No. 107754/Z/15/Z) and the UK government. The views expressed in this publication are those of the authors and not necessarily those of other parties mentioned in this publication." We note that one or more of the authors have an affiliation to the commercial funders of this research study: Gilead Sciences Inc.

3.1. Please provide an amended Funding Statement declaring this commercial affiliation, as well as a statement regarding the Role of Funders in your study. If the funding organization did not play a role in the study design, data collection and analysis, decision to publish, or preparation of the manuscript and only provided financial support for research materials. in the form of authors' salaries and/or research materials, please review your statements relating to the author contributions, and ensure you have specifically and accurately indicated the role(s) that these authors had in your study. You can update author roles in the Author Contributions section of the online submission form. Please also include the following statement within your amended Funding Statement. “The funder provided support in the form of material support for salaries for authors [insert relevant initials] but did not have any additional role in the study design, data collection and analysis, decision to publish, or preparation of the manuscript. The specific roles of these authors are articulated in the ‘author contributions’ section.” If your commercial affiliation did play a role in your study, please state, and explain this role within your updated Funding Statement.

**Response: The authors have modified the funding statement to "Funding for the study to SM and DK was provided as Investigator Sponsored Research (ISR-17-10142) from Gilead Sciences Inc., Foster City Ca. Gilead Sciences which did not play a role in the study design, data collection and analysis or decision to publish. DI and MB are employees of Gilead Science and critically reviewed the manuscript but did not play a role in the decision to publish. TM and RM received support from the Fogarty International Centre of the National Institutes of Health (D43TW010547). TM was supported (research materials plus salary) by DELTAS Africa Initiative SSACAB (Grant No. 107754/Z/15/Z) of the African  Academy of Sciences (AAS) Alliance for Accelerating Excellence in Science in Africa (AESA), New Partnership for Africa’s Development Planning and Coordinating Agency (NEPAD Agency) and the Welcome Trust (Grant No. 107754/Z/15/Z) and the UK government, See highlighted in manuscript lines 394-403.**

3.2. Please also provide an updated Competing Interests Statement declaring this commercial affiliation along with any other relevant declarations relating to employment, consultancy, patents, products in development, or marketed products, etc.  Within your Competing Interests Statement, please confirm that this commercial affiliation does not alter your adherence to all PLOS ONE policies on sharing data and materials by including the following statement: "This does not alter our adherence to  PLOS ONE policies on sharing data and materials.” (as detailed online in our guide for authors http://journals.plos.org/plosone/s/competing-interests). If this adherence statement is not accurate and there are restrictions on sharing of data and/or materials, please state these. Please note that we cannot proceed with consideration of your article until this information has been declared.

**Response: The authors have provided an updated Competing Interests Statement declaring this commercial affiliation along with any other relevant declarations relating to employment, consultancy, patents, products in development, or marketed products, etc. The authors also provided an amended Funding Statement declaring this commercial affiliation, as well as a statement regarding the Role of Funders in the study on the online submission forms. DI and MB are employees of Gilead sci. They have no competing interest with respect to consultancy, patents or products marketed or in development and there are no restrictions on sharing of data, all data from the study is freely available.**

**Reviewer #2 responses**

1. The authors should carefully review Table I and Figure 2 for consistency and agreement. For example, Table I seems to list 202 participants with baseline VL suppression who were on first line NNRTI while Figure 2 states the number at 175.

**Response: Table 1 gives information on the 306 participants who were enrolled in this study. Of the 306 participants, 202 were suppressed on first line regimens. Figure 2 describes these 202 participants who were eventually followed in 2018. Thus, out of the 264 participants who were followed, 175 (68.1%) were suppressed on first line ART regimens.**

2. Line 168: it is of interest for the reader to know how well attended were the bimonthly counselling and outreach sessions

**Response: The bimonthly counselling and outreach sessions were amazingly well attended by most of the participants and their care givers.**

3. Forty-two baseline participants were not in the follow-up cohort. Were their characteristics similar or different from the follow-up cohort?

**Response: A revision has been made to line 229-235. We evaluated the characteristics of the 42 participants who were not included in the follow-up and found that only age was significantly associated with transferring or loss to follow up. Participants who were at least 15 years of age were more likely to transfer their care or lost to follow up.**

**4.** Viral load testing did, indeed, appear to be beneficial for care. However, it was not used as the sole criteria for switching, as some baseline VL suppressors switched, and some baseline VL failures stayed on their original first line assignment. The authors might consider making this point in the discussion.

**Response: The authors agree with the reviewer that the viral load testing was not the sole criteria for switching. In the discussion (lines 308-316), we note that PI switching for virologic failure was not uniform although most (83%) of first line failures were switched and only 17% were not.**

5. It appears that this cohort experience emphasizes the concept of a ’poor adherence phenotype’ (baseline VL failure with substantial post-switch VL failure) and a ‘good adherence phenotype’ (with baseline VL suppression, likely to remain suppressed no matter whether maintaining first line or switching).

**Response: We agree that baseline VL suppression predicted follow-up suppression no matter whether maintaining first line or switching. However we did not capture individual adherence measures, although adherence counselling was provided at each 2 monthly visit as drugs were dispensed, Thus as the reviewer notes, we found that baseline viral load suppression is the most significant, independent predictor of VL suppression at follow-up as shown in Table 3 and in line 280-289.**

6. Table 2/CD4: while the numbers listed are likely accurate, it is not clear that they are what the reader might be interested in. One would like to know how the CD4 counts were distributed in those with VL suppression s VL failure. It appears that not all subjects have CD4 data available. For example, among the 215 VL suppressors, there are only 127 listed with values for CD4. This can be somewhat confusing and bears clarification.

**Response: We agree with the reviewer that this is confusing. In fact, 41% of the CD4’s for participants were missing. Thus, this variable was excluded from the multivariate model. We have indicated missingness as a footnote to Table 2.**

1. It is disturbing to see the authors classifying themselves into two separate groups that, though *apart* for unspecified reasons, nevertheless state each had contributed *equally* to the manuscript: Are the unspecified contributions of the first-named group (line 27) ‘*separate but equal*’ to those of the second-named &group (line 28) and therefore identical with the once legal principle of racism and discrimination in the United States ? That principle has been discredited. Or is the first-named group claiming to be ‘*more equal*’ than the second-named & group, reminiscent of Orwell’s *Animal Farm* ? Disturbingly, not a single one of the authors in the first-named group states that their primary affiliation is anchored at an institution in Zimbabwe. In the second-named &group of 10 authors, by contrast, those with Zimbabwean names and / or institutional affiliations predominate by a ratio of 9/1. This separation into two classes of authors is evident on the title page. The authors must find a way to put an end to such apartheid. I suggest they follow PLOS convention and specify select categories of contributions, e.g. similar to the ones in ref. [2]:

**Response: The authors have rearranged and specified the authors contributions as per PLOS one guidelines. Number 1 being the main author and other co-authors. The authors contribution was summarised as in table below:**

**Table 1: Authors contributions**

| **Contributor Role** | **Authors** |
| --- | --- |
| **Conceptualization** | **TM, DK, RM** |
| **Data Curation** | **TM, RM** |
| **Formal Analysis** | **TM RM** |
| **Funding Acquisition** | **DI, MB** |
| **Investigation** | **CM, TS, VK, JM^6,7^** |
| **Methodology** | **DK, RM** |
| **Project Administration** | **KM, SM, JM^4^** |
| **Resources** | **KM, DK, SM,** |
| **Software** | **TM RM** |
| **Supervision** | **SM, KM** |
| **Validation** | **JM^6,7^, JM^4^** |
| **Visualization** | **MM** |
| **Writing – Original Draft Preparation** | **DK, TM, VK, RM** |
| **Writing – Review & Editing** | **DI, MB** |

**JM^6,7^ - Justen Manasa , JM^4^- Junior Mutsvangwa**

2. The title of PONE-D-20-20773 specifically states that this is a “*prospective longitudinal study*” that ran from 2016 to 2018. On close reading, in particular of the Methods section on ‘*HIV VL testing*’, it appears that the 2018 segment of the study rests entirely on data obtained as part of CBART (Community Based Virus Load Differentiated Care in Rural Africa). But CBART did not exist in 2016, the initial year of the findings reported in PONE-D-20-20773. How then might it be possible that a two-year “*prospective longitudinal study*” is IRB-approved in 2016, at its inception, yet uses clinical protocol and data of CBART, which started years later, on February 1, 2018. From a 2016 IRB perspective, CBART represents an extrinsic and later protocol. This fact, in turn, might raise the added complication of post-hoc analysis of data, up to and including the possible prospect that PONE-D-20-20773 itself could, from its 2016 start, not be a strictly true and genuine “*prospective longitudinal study*”.

The Methods section on ‘*Ethics approval and consent to participate*’ does neither address nor resolve the conundrum of how the 2018 CBART dovetails with the 2016 IRB approval of PONE-D-20-20773. Rather, the formal introduction of two different IRB approvals - “*by the Medical Research council of Zimbabwe (MRCZ/A/2269) and by the* *Biomedical Research Training Institute IRB (AP143/2018)*” – further enhances this conundrum: Where participants enrolled in MRCZ/A/2269 **and** in AP143/2018 ?

Did all the patients / parents sign two different assent /consent forms?

No only the consent form signed is for the MRCZ approved study

An attentive reader is left with the impression that PONE-D-20-20773 might have been skilfully tiled and assembled from different protocols

If this should be so, this fundamental ethics issue might be resolved properly by, and might require, an IRB approval for human data use.

To address this matter objectively, I suggest that the currently unavailable and inaccessible IRB approvals MRCZ/A/2269 and AP143/2018 are uploaded to the PLOS ONE submission site for review. They are not disclosed at this time, just cite

Such an IRB approval for ‘data tiling and compiling’ will leave another conundrum unresolved, however: The CBART study, which supposedly completed in February 2020, specifically defines the identification of “*drug resistance mutations*” as one of its two Secondary Outcome Measures, the other one being the “*Number of participants with* *confirmed virology failure who switched regimens. Change to second line regimen after* *confirmed virological failure*” (see: <https://clinicaltrials.gov/ct2/show/NCT03986099>). Yet PONE-D-20-20773, which rests on that very CBART study, states “*that drug* *resistance testing was not performed on patients failing ART”* (line 351). The authors themselves instantly recognize this to be “*a limitation of the study”* (line 350). The authors leave utterly unmentioned that mutation analysis is integral to CBART. This raises questions: Why do they not use CBART data on “*drug resistance mutations*”, available at least per protocol, in a manner that renders PONE-D-20-20773 as strong scientifically as it could and should be? Are certain CBART data too good for PONE-D-20-20773? Is it really the authors’ choice to submit to PLOS ONE a manuscript for publication that, in their words, is weakened by their own choosing? The authors must resolve these conundrums.

**Response: The authors agree with the reviewer and apologize for the word prospective. The CBART Study indeed started in 2018 and assent/consent was obtained from the participants to extract their past (2016) clinical records. The authors have removed the word Prospective in the study design and have revised the ethical approval section for better clarity (See highlighted line 201-205). This specific paper focuses on the baseline Viral load (2016 Baseline viral load) of the enrolled participants, data on objectives of the CBART study are still being curated.**

**The IRB approval from the BRTI -*AP143/2018* is an institutional review that precedes the consent and enrolment form MRCZ/A/2269, the national regulatory and ethical IRB which approved the study. The study was first approved by the institution before it proceeds to the national regulatory and ethical board.**

**The conundrum is an important question, with a simple answer. The CBART study is a randomized trial of POC and SOC virus load testing, with the secondary endpoints (rate of drug switching and resistance testing). PONE-D-20-20773 includes only the CBART enrolment virus load data obtained between Feb 2018-and September 2018. The full CBART study (see:** [**https://clinicaltrials.gov/ct2/show/NCT03986099**](https://clinicaltrials.gov/ct2/show/NCT03986099)**) includes resistance testing of the prospectively enrolled and followed “confirmed virologic failures” as noted by the reviewer. The period in the longitudinal study PONE-D-20-20773, did not include resistance testing and this is recognized as limitation of the longitudinal study (lines 356-357). “A limitation of the study is that drug resistance testing was not performed on patients failing ART”**

3. Per the WHO HIV Treatment Guidelines and academic publications (see [2]), a core aspect of viral load-differentiated care is to “both serve the needs of PLHIV (*people* *living with HIV*) better and reduce unnecessary burdens on the health system” [1], to “reduce the frequency of clinic visits for patients stable on ART” with “anticipated reductions in the costs of clinic visits, due to these being less frequent for many patients”[2]. Consequently, “differentiated care models could decrease health systems costs in 38 countries in sub-Saharan Africa” [3]. This decisive aspect of viral load-differentiated care is entirely absent from PONE-D-20-20773, and its focus on just viral load management makes the study almost a description of routine clinical decisions – clearly, every doctor entrusted with the care of HIV patients will adjust the ARV regimen in response to viral load. By focusing on viral load-differentiated care, the authors have given themselves the burden of showing more than just viral load responses, and they have to answer simple questions like ‘*Did the patients who responded to viral load-differentiated care in actual* *fact require fewer clinic visits, fewer medical resources, and did they have fewer days lost* *at school, fewer hours lost on the way to clinic, fewer hours wasted while waiting to be* *seen by the medical team ?* ‘If the authors can create a clinic-based quantitative monetary parameter, even better (e.g. costs, ‘*how many bandages used?* ’, etc.)

I respectfully submit it might be possible to read some of the answers to such questions, essential to viral load-differentiated care, off the medical charts of enrolled patients. The inclusion of such data will turn this manuscript into a reality-based contribution to the discussion on resource utilization in viral load-differentiated care. I encourage the authors to make that effort.

**Response: The authors agree with the reviewer, this paper focuses on the 2016 baseline characteristics and follow-up VL of the participants enrolled in the CBART protocol in 2018. For PONE-D-20-20773, the authors have removed the word “differentiated” VL throughout the manuscript as the management of participants was ecological and treatment decisions were made by nurse-providers at Chidamoyo Hospital.**

4. I note a number of the usual diligence issues that reviewers like to discover as evidence of their own diligence, such as spelling and capitalization (e.g. line 10: ‘Cape **t**own’), bringing references up to date and into PLOS format (e.g. ref. 12 is one of many that lack the specified format), improving the pixilation of images (e.g. Fig. 1 lacks the proper resolution), and clarifying graphics (e.g. Fig. 2: Why were patients with VL < 1000 switched to 2nd line ? Why were patients with VL ≥ 1000 maintained on 1st line?).

**Response: Cape Town line 10 was corrected. All the references including reference 12 were revised and updated according to PLOS ONE reference format. The pixilation of images was also improved. With regard to figure 2, the changes in regimens were identified in 2017 through chart review. We have attached a figure legend which includes:**

**Longitudinal assessment of VL measures at baseline and after 18 months of follow-up among 264 children and adolescents. The regimen changes (from NRTI to PI-based combinations) were based on the Zimbabwe guidelines on formulations, dosing, toxicities, and availability of combination ART in 2017.**
