# Supplementary material for: Viral load care of HIV-1 infected children and adolescents: A longitudinal study in rural Zimbabwe
Source: PLoS One. 2021 Jan 14;16(1):e0245085. doi: 10.1371/journal.pone.0245085 (PMC7808638; doi:10.1371/journal.pone.0245085)
Supplement: Attachment — Submitted filename: Response to reviewers comments.docx [file pone.0245085.s005.docx]

**PONE-D-20-20773-R1**

Second review of Tichaona Mapangisana et al.

**Viral load care of HIV-1 infected children and adolescents: a longitudinal study in rural Zimbabwe.**

The authors’ revisions are sincerely appreciated.

Before proceeding, I wish to respectfully submit to the authors to kindly consider that with their decision to submit to PLOS ONE, their paper is no longer directed to a specialty audience, as was their presentation to the 11th International Workshop on HIV Pediatrics in July last year on which their manuscript is based (Abstract #51, http://regist2.virology-education.com/abstractbook/2019/abstractbook_Pediatrics2019.pdf ). They now have elected to place themselves onto a different and much elevated stage. In order to earn the full impact that this stage offers and that their effort deserves, they need to leave the previous small format and its specialty language well behind, they need to raise their voice, need to argue additional context and attract the attention of a much wider public with clarity, diligence, and precision. I encourage them to re-examine their manuscript from the much more global perspective of a much more global audience. In that contact, I wish to advance the following suggestions for their consideration:

**1.** Foremost, the authors must put the Chidamoyo Hospital data documenting “*suppression increased from 68% in 2016 to 81% in 2018* ” into a context that a non-African and non-HIV specialist audience understands and can appreciate. Writing “*Whole blood 154 samples were transported to Harare (300 km one-way trip)*” is just not enough and invites imagery of car rides on autobahn and interstate, at least

of driving on asphalted roads ... Not so.

The authors **MUST** detail the setting of that hospital - the next major asphalted road is hours away, the remote rural population it serves is economically so disenfranchised that wearing of soled leather shoes is unheard of, per capita income is even less than the $2000 annual that 50% of Zimbabweans earn (https://www.averagesalarysurvey.com/zimbabwe, https://en.wikipedia.org/wiki/Economy_of_Zimbabwe). In fact, just a few months ago one of the authors was forced at gun point to open the safe of Chidamoyo Hospital at 4 AM and surrender the institution’s opulently rich cash reserve of USD $1700 – to a dozen desperately impoverished soldiers of the Zimbabwe National Army (ZNA) who, when caught by police and handed back to the ZNA, promptly found the military’s cover and support (https://www.zimbabwesituation.com/news/12-armed-robbers-arrested-after-robbing- chidamoyo-mission-hospital/ **;** http://newsofthesouth.com/new-twist-to-chinhoyi-zna- officers-armed-robbery-case/ ).

I respectfully submit that the degree of poverty in the authors’ study population is beyond the imagination of the very most readers of PLOS ONE. As is the extent of HIV in the study population; and likewise the extent of tuberculosis in consequence of HIV. Under such conditions, to achieve “*suppression increased from 68% in 2016 to 81% in 2018* ” in a population of economically marginalized adolescents in the remote hill country of Northern Zimbabwe is a most extraordinary achievement in the fight against a global vial epidemic; even more so is the data collection and the scientific analysis of the authors - - not just 1

**PONE-D-20-20773-R1**

relevant in the HIV context, but also relevant in the tuberculosis context, an aspect that **MUST** be introduced in the Discussion (and yes, Chidamoyo Hospital also treats HIV-TB and TB patients). Making pills to fight HIV globally and making vaccines to fight SARS-Cov-2 globally has no effect on these viral pandemics. For effect, we need institutions like Chidamoyo Hospital. For effect, we need nurses, physicians, and scientists exactly like these authors.

Their voice must be heard. I encourage the authors not to hesitate in making the explicit connection to SARS-Cov-2 in their Discussion – who else is there to administer the SARS-Cov-2 vaccines and to capture the data of that vaccine’s effect but nurses, physicians, and scientists like them? Their manuscript is not just HIV health care delivery, it is implicitly a model of anti-viral health care delivery where it counts most – no one is safe until we all are. The authors should consider that SARS-Cov-2 is anticipated to devastate Zimbabwe (https://www.un.org/africarenewal/news/coronavirus/covid-19-could-prove-“disastrous”- zimbabwe-undp-study-finds ; https://allafrica.com/stories/202010230244.html ; https://allafrica.com/stories/202010080945.html ). They have proven that they can deliver anti-viral care under conditions of extreme poverty, and they should spell it out. A few low-key sentences in the Discussion, with pointers already in the Introduction, are sufficient.

**Response: Thank you very much. The authors share the reviewer’s enthusiasm for all the work done by the Chidamoyo Christian hospital. The authors have added description of the impoverished circumstances in rural Zimbabwe specifically the extreme poverty as described by the National Zimbabwe statistical Center. See highlighted under study setting lines 163-166.**

**The authors have added a brief summary of the medical services provided by Chidamoyo Christian Hospital. See highlighted under study setting lines 165-172 and a link to a video presentation on Chidamoyo.**

**We thank the reviewer for highlighting the findings important to HIV care in a disadvantage population. We have added the sentence “We found that, suppression increased from 68% in 2016 to 81% in 2018 in a population of economically marginalized children and adolescents in an impoverished rural community in Mashonaland West province”. See highlighted in the discussion section lines 329-331.**

**In the third paragraph, we have emphasized on the economic and logistic challenges in delivering ART in rural communities. See highlighted lines 349-359. This paragraph points out the challenges of transport, distance and basic infrastructures.**

**2.** The map on the location of the hospital and the area it serves still is far from optimal and not meeting PLOS standards. May I be allowed to offer the authors a graphical proposal (attached). The approximate location of Chidamoyo Hospital is indicated by the white star; the relative locations of the five outpatient sites Batanayi (not ‘Batanai’ !), Magororo, Chedope, Nyamutora, and Zvarai are indicated by white circles as transcribed from the cartographic identifiers provided in https://www.humanitarianresponse.info/sites/www.humanitarianresponse.info/files/ZWE_ MashWest_Province_A0_v1.pdf .

**Response: The authors appreciate the value of the attached figure and have included this rather than the original figure. Thank you very much.**

**3.** Please aim for an abbreviation-minimized reading experience. Remove from the text (not the tables) whenever possible any HIV specialist letter salad and alphabet soup like VL, NNRTI, 3TC, EFV, TDF/3TC/EFV and even TDF +3TC +NVP – please consider typing out the actual names - - you write for a broad audience! Please check again spelling infelicities and like imprecisions. PLOS does not do text editing and proof-reading, that is your responsibility! Please be diligent and consistent to the extreme – it is either “*second-line”* or “*second line”!*

**Response: Thank you for this. We have minimized abbreviations throughout the manuscript and be consistent with hyphenation of second-line.**

**4.** In the Introduction the statement of the Results “*switched to second-line boosted PI ART with abacavir*” clashes with the immediately following statement in the Conclusion “*switching to second line ritonavir boosted PI-based ART*” – what is it: abacavir or ritonavir? This clash is repeated later in the text. Please address and resolve.

I suggest one more round of review. Hartmut M. Hanauske-Abel, MD PhD.

**Response: Thank you. The reviewer should note that, second-line protease inhibitor (PI)-based regimens are always “boosted” meaning that they are co-formulated with a low dose of ritonavir. Abacavir and lamivudine are nucleotide reverse transcriptase inhibitors included in second-line regimens. We have resolved this ambiguity in the abstract and throughout the manuscript. See highlighted lines 64 and 67-68.**
